# Supplementary material for: Content-rich biological network constructed by mining PubMed abstracts
Source: BMC Bioinformatics. 2004 Oct 8;5:147. doi: 10.1186/1471-2105-5-147 (PMC528731; doi:10.1186/1471-2105-5-147)
Supplement: Additional File 2 — The original results of the above study (non-essential files are deleted to keep the file size under the limit set by BMC bioinformatics). [file 1471-2105-5-147-S2.bz2 › chilibotAdditionalFile2/dip20/141ID7739537E501/html/TFDP1_RB1.html]

 


 **TFDP1** and **RB1** 
  
Found 66 abstracts in PubMed, retrieved 20.  
 

 What does Google say? 
 PDF only 
| .edu only 

---

**Interactive relationship** (e.g. stimulation, inhibition, etc)

**Inhibitory relationship**- Co expression of the retinoblastoma  [ **RB1** ]  gene product RB abolishes baseline E2F 1 DP 1  [ **TFDP1** ]  transcriptional activity as well as TRIP Br KRIP 1 co activation, both of which are restored by the adenovirus E1A oncoprotein.  Ref: 11331592 EMBO J, 2001
- Although the mdm2 gene is thought to act as an oncogene by blocking the activity of p53, recent studies indicate that mdm2 can act independently of p53 and block the G1 cell cycle arrest mediated by members of the retinoblastoma  [ **RB1** ]  gene family and can activate E2F1 DP1  [ **TFDP1** ]  and the cyclin A gene promoter.  Ref: 9858609 Mol Cell Biol, 1999
**Neutral relationship**

**Non-interactive relationship** (e.g. studied together, co-existance, homology, etc.)

- Because Cdk4 6 phosphorylates retinoblastoma  [ **RB1** ]  protein pRb family members that then modulate the transcriptional activity of E2F DP1  [ **TFDP1** ]  complexes, we examined the involvement of these components in DNA damage evoked neuronal death.  Ref: 10777774 J Neurosci, 2000
- Release of E2F1 DP1  [ **TFDP1** ]  heterodimers from repression mediated by the retinoblastoma  [ **RB1** ]  tumor suppressor pRB triggers cell cycle entry into S phase, suggesting that E2F1 and DP1  [ **TFDP1** ]  proteins must act in unison, either to facilitate or to suppress cell cycle progression.  Ref: 12588846 Development, 2003
- mdm2 has recently been shown to interact with the retinoblastoma  [ **RB1** ]  tumor suppressor protein p Rb, and the E2F 1 and DP1  [ **TFDP1** ]  transcription factors.  Ref: 9322885 Leuk Lymphoma, 1997
- We have shown that p202, an IFN inducible 52kDa primarily nuclear phosphoprotein whose expression in transfected cells inhibits cell proliferation, interacts with the retinoblastoma  [ **RB1** ]  tumor suppressor protein pRb and the transcription factor E2F E2F 1 DP 1  [ **TFDP1** ]  in vitro and in vivo.  Ref: 9233764 Oncogene, 1997
